# Association of neighbourhood socioeconomic trajectories with preterm birth and small-for-gestational-age in the Netherlands: a nationwide population-based study

**DOI:** 10.1016/j.lanepe.2021.100205

**Published:** 2021-08-24

**Authors:** Lizbeth Burgos Ochoa, Loes CM Bertens, Pilar Garcia-Gomez, Tom Van Ourti, Eric AP Steegers, Jasper V Been

**Affiliations:** 1 Department of Obstetrics and Gynaecology, Erasmus MC – Sophia Children's Hospital, University Medical Centre Rotterdam, Rotterdam, Netherlands; 2 Division of Neonatology, Department of Paediatrics, Erasmus MC – Sophia Children's Hospital, University Medical Centre Rotterdam, Rotterdam, Netherlands; 3 Erasmus School of Economics, Tinbergen Institute and Erasmus Centre for Health Economics Rotterdam, Erasmus University Rotterdam, Rotterdam, The Netherlands; 4 Erasmus School of Health Policy and Management, Erasmus University Rotterdam, Rotterdam, the Netherlands; 5 Department of Public Health, Erasmus MC, University Medical Centre Rotterdam, Rotterdam, Netherlands

## Abstract

**Background:**

Adverse birth outcomes have serious health consequences, not only during infancy but throughout the entire life course. Most evidence linking neighbourhood socioeconomic status (SES) to birth outcomes is based on cross-sectional SES measures, which do not reflect neighbourhoods’ dynamic nature. We investigated the association between neighbourhood SES trajectories and adverse birth outcomes, i.e. preterm birth and being small-for-gestational-age (SGA), for births occurring in the Netherlands between 2003 and 2017.

**Methods:**

We linked individual-level data from the Dutch perinatal registry to the Netherlands Institute for Social Research neighbourhood SES scores. Based on changes in their SES across four-year periods, neighbourhoods were categorised into seven trajectories. To investigate the association between neighbourhood SES trajectories and birth outcomes we used adjusted multilevel logistic regression models.

**Findings:**

Data on 2 334 036 singleton births were available for analysis. Women living in stable low-SES neighbourhoods had higher odds of preterm birth (OR[95%CI]= 1·12[1·07-1·17]) and SGA (OR[95%CI]= 1·19[1·15-1·23]), compared to those in high SES areas. Higher odds of preterm birth (OR[95%CI]= 1·12[1·05-1·20]) and SGA (OR[95%CI]=1·12[1·06-1·18]) were also observed for those living in areas declining to low SES. Women living in a neighbourhood where SES improved from low to medium showed higher odds of preterm birth (OR[95%CI]= 1·09[1·02-1·18]), but not of SGA (OR[95%CI]= 1·04[0.98-1·10]). The odds of preterm or SGA birth in other areas were comparable to those seen in high SES areas.

**Interpretation:**

In the Netherlands, disadvantaged neighbourhood SES trajectories were associated with higher odds of adverse birth outcomes. Longitudinal neighbourhood SES measures should also be taken into account when selecting a target population for public health interventions.

**Funding:**

Erasmus Initiative Smarter Choices for Better Health.

**Research in context**

**Evidence before the study**

We searched PubMed and MEDLINE databases for literature published in any language before December 1, 2020, using the following search terms: “neighbourhood” AND “trajector* OR change* OR histor*” AND “socioeconomic OR social OR economic OR poverty OR deprivation” AND “birth outcome* OR preterm birth OR prematur* OR small-for-gestational-age OR birth weight”. We identified three relevant studies. The first study was conducted in California (US) and used the Maternal Infant and Health Assessment survey (2003-2009, N=23 291). Their study investigated the association between longitudinal neighbourhood poverty trajectories and preterm birth. Compared to births from long-term low poverty neighbourhoods, those from areas with long-term high or increasing poverty had 41% and 37% increased odds of preterm birth, respectively. The second study used Texas (US) birth certificate data (2009-2011, N=470 896) to examine the association between longitudinal measures of neighbourhood poverty and adverse birth outcomes, i.e., preterm birth, low birth weight and small-for-gestational-age (SGA). They found that long-term high and moderate poverty histories, along with increasing and decreasing poverty were associated with higher odds of adverse birth outcomes. Both studies used an extensive period (20 and 40 years) to construct the neighbourhood trajectories. Last, a study conducted in New York City explored the association between living in a gentrifying neighbourhood and birth outcomes (2008-2010, N=126 165). They found that, for disadvantaged groups, living in rapidly gentrifying neighbourhoods was associated with increased incidence of preterm birth.

**Added value of this study**

Based on data from the nationwide perinatal registry in the Netherlands (2003-2017, N= 2 334 036), we investigated the association between trajectories of neighbourhood socioeconomic status (SES) and adverse birth outcomes, i.e. preterm birth and SGA. Our study shows that, in the Netherlands, adverse neighbourhood SES trajectories were associated with higher odds of adverse birth outcomes. Women living in persistently low SES areas or areas that declined to low SES had higher odds of preterm and SGA birth than women living in the most advantaged areas. Also, living in a neighbourhood whose SES shifted from low to medium was associated with higher odds of preterm birth, but not SGA. Importantly, the odds of preterm or SGA birth in other areas were comparable to those seen in high SES areas. To our knowledge, this is the largest study to have investigated the relationship between neighbourhood SES trajectories and birth outcomes, and the first to use short-term changes in neighbourhood SES.

**Implications of all available evidence**

Our results indicate that, in the Netherlands, disadvantaged neighbourhood SES trajectories were associated with higher odds of adverse birth outcomes. Results from this study suggest that longitudinal neighbourhood SES measures should also be taken into account when selecting a target population for public health interventions.

Alt-text: Unlabelled box

## 1 Introduction

Adverse birth outcomes, defined here as preterm birth and small-for-gestational-age (SGA), have serious health consequences, not only during infancy but throughout the entire life course [1]. Being born preterm or SGA increases the risk for early-life mortality, and subsequent lifelong morbidity [2,3]. Evidence from population-based studies has consistently linked low neighbourhood socioeconomic status (SES) with adverse birth outcomes, even after adjustment for individual characteristics [4,5]. As such, adverse birth outcomes could be considered the earliest manifestations of socioeconomic inequalities. The majority of the current literature is based on cross-sectional measures of neighbourhood SES [6]. Cross-sectional measures fail to reflect that neighbourhoods are not static but dynamic entities that can experience improvement or deterioration as the result of economic, social and migration processes [7,8].

Longitudinal approaches to investigating the link between neighbourhood conditions on health outcomes are scarce. The best available evidence comes from social experiments, e.g., Moving to Opportunity (MTO), a program that randomised disadvantaged families in the US to receive vouchers for residential mobility. People who moved to low poverty neighbourhoods within MTO experienced improvements in various health outcomes [9]. However, studies like MTO investigated only the effects of improving neighbourhood SES, while it is also relevant to look at the consequences of negative changes. From a policy and public health perspective, it is essential to explore changes in neighbourhoods’ SES themselves, as the majority of the population does not move, or when they do, it is generally to similar areas [10].

Few studies have investigated the association between the change in neighbourhood socioeconomic characteristics and birth outcomes [6,7,11]. These available studies are based on single US states and have relatively limited sample sizes. Their findings may not apply to European countries due to demographic, social, economic, and health care differences [10]. Most of these studies place their focus on long-term neighbourhood change (e.g. across 40 years). However, exploring the link between short-term changes and health is also relevant. One of the main mechanisms through which changes in neighbourhood SES may affect health outcomes is stress [6,7]. It has been argued that neighbourhood residents are probably accustomed to a certain amount of perks and problems within their neighbourhood, and it might be the rapid changes that result in health impact [12]. Short-term changes in neighbourhood SES have been associated with changes in risks factors for adverse birth outcomes, e.g. unhealthy food environment [13], and poor mental health [14]. Only one previous work, conducted in New York City, has explored the association between short-term changes in neighbourhood SES and birth outcomes [11]. However, this study only focused on gentrifying neighbourhoods rather than the full spectrum of SES trajectories.

The Netherlands offers an ideal setting for the study of short-term changes in neighbourhood conditions and their association with birth outcomes. Previous research has shown that a fifth of the Dutch neighbourhoods experienced decline or improvement in four years [15]. Additionally, the country is investing in policies that aim to improve neighbourhood SES in the short term [16]. The purpose of this study is to describe the association between short-term neighbourhood SES trajectories and birth outcomes in the Netherlands. Based on the available literature, we hypothesise that neighbourhoods with persistently low SES, or those that decline to low SES, will show the poorest outcomes [6,7,11,17].

## 2 Methods

### 2.1 Study design and participants

In this retrospective population-based cohort study, we linked individual-level birth records to routinely collected neighbourhood-level data, population register data, and income and tax records. The cohort comprised singleton births with gestational ages between 24+0 and 41+6 weeks registered in the Netherlands between 1 January 2003 and 31 December 2017. Birth records before 2003 were not included in the analysis as information on household income is only available from 2003 onwards.

We obtained the birth records from the Netherlands Perinatal Registry (Perined). Perined comprises routinely collected data on maternal characteristics, pregnancy, delivery, and birth outcomes, covering 97% of all births in the Netherlands [18]. The data is subject to strict quality and consistency checks to ensure that only valid values of the perinatal variables are kept in the final dataset [19]. Perined also provides the four-digit postcode of the mothers’ residence.

Statistics Netherlands (CBS) performed the individual-level linkage of Perined records to the national population registry held at CBS. As a result of this linkage, CBS assigns each mother and child a unique identification number (RIN number). This identifier is a meaningless and dimensionless number that identifies a natural person [20]. Every individual in the Netherlands has a unique RIN number that is used by CBS to link a wide variety of administrative records and surveys. Given that each mother and child have unique identifiers, siblings born from the same mother are identifiable. Instances, where the linkage algorithm did not link a registered birth to a RIN number could be because the mother was not registered in the population records (non-residents), the child was stillborn, or due to linkage error (false-matches and missed-matches). Given that stillbirths were non-linkable, records available for analysis consisted of live births only. From the available Perined birth records, 3% could not be linked to CBS data.

CBS population and income and tax records include sociodemographic information of the country's residents. This information is routinely collected from different sources, e.g., municipality records and the Dutch Tax and Customs Authority. CBS data registries are subject to strict quality checks and follow several procedures to ensure the validity of the data [21].

### 2.2 Data variables and measurement

The following definitions were used for the birth outcomes: 1) preterm birth, any livebirth occurring from 24+0 weeks of gestational age and before 37+0 weeks, and 2) SGA birth, birth weight below the 10^th^ centile adjusted for gestational age and sex, according to national reference curves [22]. Gestational age is estimated by using information on the last menstrual cycle and foetal scans [23]. Births with gestational age <24+0 were not included in the analysis as Dutch national multidisciplinary guidelines advise against active management of babies born at gestational ages of less than 24 weeks and 0 days [24,25]. Furthermore, birthweight <400g was considered implausible and treated as missing, as European and national guidelines advise against the active management of babies with birthweight below this threshold [25]. Based on previous studies, birthweight was also considered implausible and set as missing if >6500g [26,27].

We used the household income corresponding to the child's year of birth to measure individual-level SES. Researchers have recommended using household income to better measure women's SES over other individual-level measures for health inequalities research [28]. Moreover, health inequalities research has shown that household income performs as good as other individual-level SES indicators (e.g. education or composite measures) in capturing health variation [29]. Information on household-equivalised disposable income was obtained from CBS income and tax records. This measure accounts for the household size and composition using the modified Organisation for Economic Co-operation and Development (OECD) equivalence scale [30]. Data on mother's education was not included in the main analysis (but in a sensitivity analysis) as information on this variable was missing for a considerable part of the dataset (i.e., 19·6%) [31].

We obtained information on maternal ethnicity and residential history from CBS records. Ethnicity was assigned based on the mother's country of birth. CBS categorises this variable based on the largest ethnic groups present in the Netherlands: Dutch background, Turkish, Moroccan, Surinamese, Antillean, others western, others non-western [32]. A woman would have a western migration background if she or at least one of the parents was born in Europe, North America or Oceania [32]. Information on whether the mother was a first-generation or second-generation migrant was also obtained from CBS records.

We used the Netherlands Institute for Social Research (SCP) Status Scores to measure neighbourhood socioeconomic status [33]. The SCP Status Scores are a relative measure of neighbourhood SES calculated for areas corresponding to four-digit postcodes, with an average of 4 000 inhabitants [34], and a median size of 5·3 km^2^. The SCP Status Scores are based on postcode-level data collected yearly by CBS, which is calculated by aggregating the information of all residents from each four-digit postcode [35]. The SCP Status Scores summarise information from four indicators: 1) average neighbourhood income, 2) percentage of inhabitants with a low income, 3) percentage of inhabitants without a paid job, and 4) percentage of inhabitants with a low education level. The SCP Status Scores have been previously used in health inequalities research in the Netherlands [36], [37], [38]. The SCP provides updated Status Scores every four years. For this work, we used the SCP Status Scores corresponding to the years 1998, 2002, 2006, 2010, and 2014.

The exposure of interest was neighbourhood SES trajectory. To construct the SES trajectories, we first created cross-sectional measures of neighbourhood SES by categorising the SCP Status Scores into Low (lowest quintile), Medium (second to fourth quintiles), and High (highest quintile). Then, by comparing two consecutive cross-sectional SES measures (e.g. 2006 vs 2002), the neighbourhoods were categorised into seven SES trajectories: 1) Stable High, 2) Stable Medium, 3) Stable Low, 4) Improving to High, 5) Improving to Medium, 6) Declining to Medium, and 7) Declining to Low. Categories portraying a drastic change in neighbourhood SES, i.e., Improving Low to High and Declining High to Low were also considered. However, such steep changes are rare in the Netherlands [15], and across the period 2003-2017, only <0·3% of the births could be assigned to any of these trajectories. These cases were thus included in the trajectories Improving to High and Declining to Low, respectively.

Birth records were grouped into four mutually exclusive periods (Table 1). The exposure (neighbourhood SES trajectory) was assigned to the births that occurred within each period, as stated in Table 1. For example, for each neighbourhood, the trajectory resulting from comparing 2006 versus 2002 cross-sectional SES measures was used as exposure for births occurring in the period 2006-2009. The corresponding neighbourhood SES trajectory was assigned to each birth using maternal four-digit postcode registered at delivery.

**Table 1:** Exposure assignment to birth records.

Exposure (neighbourhood SES trajectory)	Birth period
2002 vs 1998	1) 2003-2005
2006 vs 2002	2) 2006-2009
2010 vs 2006	3) 2010-2013
2014 vs 2010	4) 2014-2017

*First birth time period includes only 3 years instead of 4 (as later periods). Birth records before 2003 were not included in the analysis as information on household income is only available from 2003.

Due to privacy considerations, SCP does not calculate status scores for areas with less than 100 households [18]. Therefore, neighbourhood trajectories could not be assigned to births from mothers living in such areas or birth records without a postcode available. As a result, neighbourhood SES trajectory was missing for 1·5% of the records. Due to the low proportion of missing data, no data was imputed for the analyses.

### 2.3 Statistical analysis

To assess the relationships between neighbourhood SES trajectories and adverse birth outcomes, we used two-levels (level 1, births; level 2, neighbourhoods) logistic random-intercepts regression models with pooled cross-sections. The pooled cross-sections technique combines elements from time series and cross-sectional data to analyse datasets that consist of several cross-sections from the same population collected at different time points (e.g. years or periods) but where the observations do not refer to the same units [39,40]. The percentage of variation between neighbourhoods in preterm birth and SGA prevalence (intra-class correlation, ICC) was around 2% and statistically significant, supporting the decision to use multilevel models [41]. The Stable High SES trajectory, reflecting the most advantaged neighbourhoods, was used as reference.

We considered a set of potential confounders, i.e., variables that are (causally) associated with the outcome and related to the exposure but are not intermediate variables in the causal pathway between exposure and outcome (mediators) [42,43]. We adjusted the models for the following individual-level characteristics: maternal ethnicity, migration generation, maternal age at delivery in categories (≤19, 20-34, ≥35 years), equivalised household income in categories (quintiles), and parity registered at (antenatal) intake (primiparous vs multiparous). Maternal lifestyle factors (e.g. smoking, drug and alcohol use, BMI), aside from suffering from severe underreporting [44], have been suggested as mediators for the relationship between neighbourhood SES and health outcomes [45], [46], [47], [48]. To avoid bias due to over-adjustment [49,50], these variables were not included in the main models. At the neighbourhood level, we did not adjust for physical (e.g. pollution and greenness [51,52]) and social factors (e.g. social cohesion and crime [52,53]) as they have also been found to mediate the exposure-outcome relationship. Variables registering maternal comorbidities (e.g. pre-existent diabetes and hypertension) were not included in the main models as they are likely to suffer underreporting in the Perined dataset [18]

Dummy variables for all but one time period were included in the models to account (and test) for changes in the outcomes across different periods [40]. Next, interaction terms between each time-period dummy variable and neighbourhood SES trajectories were added to account for changes over time in the relationship between exposure and outcomes [40].

We conducted a set of sensitivity analyses to assess the robustness of our findings: 1) Models were additionally adjusted for the duration of residence in the neighbourhood at the time of delivery (in years). 2) We excluded births in 2006, 2010 and 2014 to assess whether our results were driven by cases born in the first years of the periods. 3) We conducted two analyses to assess the impact of women moving to a different neighbourhood during, or before, their pregnancy on the results: a) including only women who had been living in the neighbourhood for at least one year at the time of delivery, and b) restricting the analysis to women who have resided in the same residential address throughout the entire four years period corresponding to the assigned exposure (see Table 1). 4) We assessed the robustness of our results to the adjustment for the mother's educational level (low, medium, high). 5) We also assessed the robustness of our results to the adjustment for a) maternal comorbidities (pre-existing diabetes and hypertension), and b) unhealthy lifestyle factors, i.e., smoking, alcohol consumption, and drug use (binary variables). The attenuation of the estimates after adjusting for lifestyle factors may be a sign of mediation. 6) Restricted the analysis to only spontaneous births. 7) Excluded observations with implausible birthweight given gestational age values, i.e., birthweight was assumed missing if it was recorded as >1500g and gestational age <29 weeks. For gestational age 29 to 33 weeks birthweight was assumed missing if it was recorded as >2800 g [54]. 8) We fitted joint regression models for correlated binary outcomes to account for any potential interdependence between the outcomes. We followed the procedure developed by Ghebremichael [55], which was applied to a multilevel scenario by Di Fang et al [56]. 9) We conducted a siblings-comparison analysis (within-family or family fixed-effects analysis) to reduce unobserved confounding at mother's level. The siblings-comparison analysis controls by design for all time-constant (shared by the siblings) observed and unobserved confounders including the mother's ability, genetics, ethnicity, etc. The siblings-comparison model was additionally adjusted for time-variant covariates, i.e., maternal age, household income, and parity. 10) For the adjusted results that were found to be significant, we performed an analysis to assess how strong an unmeasured confounder would have to be to explain away an observed exposure–outcome relationship, i.e., E-value computation [57]. The E-value quantifies the minimum strength of association on the OR scale that an unmeasured confounder must have with both the exposure and outcome, while simultaneously considering the measured covariates, to negate the observed exposure–outcome association [57].

For all analyses, a p value of less than 0·05 was used to indicate statistical significance. All analyses were performed using R version 3.6.3 [58].

#### 2.3.1 Ethical considerations

According to Dutch law (WMO) no formal ethical review was required. According to standard procedures and under strict conditions that were fulfilled, CBS anonymised the data before making it available to the researchers [26]. Perined provided approval (19.13) for this research project.

#### 2.3.2 Role of the funding source

The funder of the study had no role in study design, data collection, data analysis, data interpretation, or writing of the report. The corresponding author had full access to all the data in the study and had final responsibility for the decision to submit for publication.

## 3 Results

**Fig. 1:**
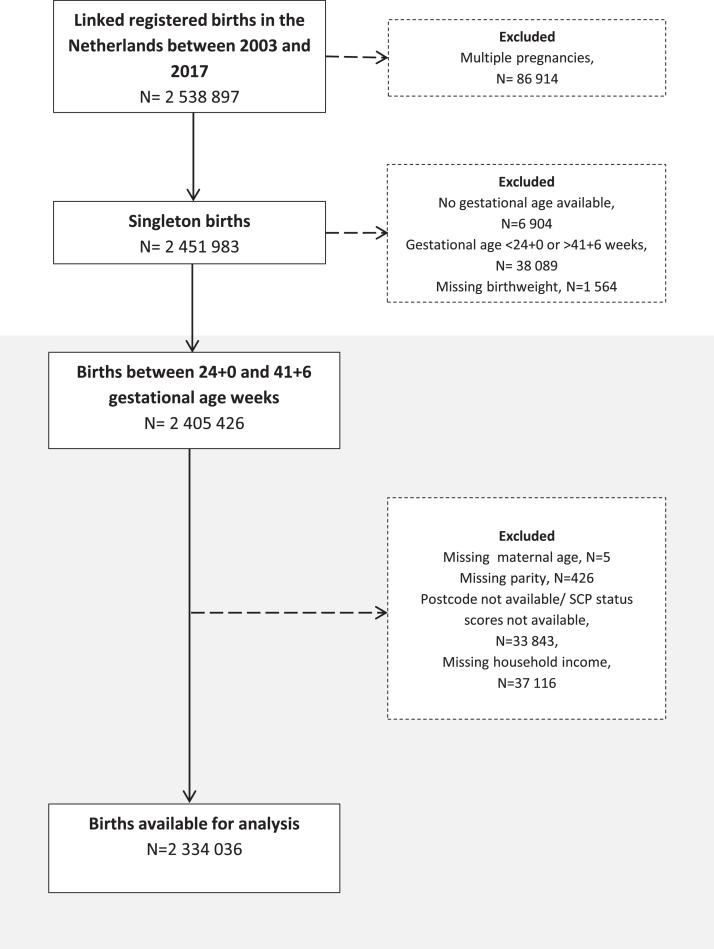
Study population flow diagram.

Between 2003 and 2017, 2 629 207 births were registered in the Netherlands, of which 2 538 897 (∼97%) could be linked by CBS. After removing multiple births, births with gestational age below 24+0 weeks or above 41+6 weeks, and births with missing data on covariates, 2 334 036 births were available for the analysis (Fig. 1).

During each of the periods, roughly one-fifth (19.5%) of the neighbourhoods saw a change in their SES, while most of the areas (80·5%) remained stable (Supplementary Figure 1). The mean maternal age was 30·6 (SD 4·8), it was at its lowest in Stable Low areas (29·6, SD 5·3), and highest in Stable High (31·8, SD 4·4) (Table 2). Stable Low areas showed the highest percentage of women with a migration background. The lowest and highest household incomes were observed in Stable Low and Stable High neighbourhoods, respectively. In terms of birth outcomes, Stable Low and Declining (Medium to Low) neighbourhoods had the highest prevalence of preterm and SGA births, while Stable High areas showed the lowest prevalence.

**Table 2:** Population characteristics of singleton pregnancies between 2003 and 2017 by neighbourhood SES trajectory.

		Neighbourhood SES trajectory						
	Total	Stable High	Stable Medium	Stable Low	Improving to High	Improving to Medium	Declining to Medium	Declining to Low
**N (% of total)**	2 334 036	359 525 (15·4)	1 054 271 (45·2)	502 546 (21·5)	91 684 (3·9)	103 483 (4·4)	108 282 (4·6)	114 245 (4·89)

	**Characteristics**							

**Maternal age, mean (SD)**	30·6(4·8)	31·8 (4·4)	30·5 (4·7)	29·6 (5·3)	31·5 (4·5)	30·4 (4·9)	31·0 (4·6)	30·0 (4·9)
**Primiparous, N(%)**	1 053 956 (45·2)	155 847 (43·4)	473 158 (44·9)	231 276 (46·0)	40 771 (46·1)	50 823 (49·1)	47 818 (44·2)	52 463 (46·1)
**Dutch**	1 707 011 (73·1)	280 970 (78·2)	853 862 (81·0)	257 874 (51·3)	72 390 (79·0)	74 170 (71·7)	86 226 (79·6)	81 519 (71·4)
**Moroccan**	99 679 (4·3)	6 850 (1·9)	24 708 (2·3)	55 031 (11·0)	1 623 (1·8)	4 066 (3·9)	2 429 (2·2)	4 972 (4·4)
**Turkish**	80 503 (3·4)	5 743 (1·6)	18 856 (1·8)	45 084 (9·0)	1 149 (1·3)	3 422 (3·3)	1 625 (1·5)	4 624 (4·0)
**Suriname**	59 851 (2·6)	8 312 (2·3)	14 644 (1·4)	26 994 (5·4)	1 997 (2·2)	2 727 (2·6)	2 200 (2·0)	2 977 (2·6)
**Antillean**	27 572 (1·2)	2 680 (0·7)	7 587 (0·7)	12 933 (2·6)	674 (0·7)	1 300 (1·3)	839 (0·8)	1 559 (1·4)
**Other non-western**	141 916 (6·1)	17 205 (4·8)	46 394 (4·4)	54 610 (10·9)	4 087 (4·5)	6 796 (6·6)	4 782 (4·4)	8 042 (7·0)
**Other western**	217 504 (9·3)	37 765 (10·5)	88 220 (8·4)	50 020 (10·0)	9 764 (10·6)	11 002 (10·6)	10 181 (9·4)	10 552 (9·2)
**Second generation**	227286 (9·8)	34 111 (9·5)	76 520 (7·2)	77 695 (15·4)	8 057 (8·8)	10 542 (10·2)	8 920 (8·2)	11 441 (10·0)
**Household income €, median (IQR)**	22 140 (14 279)	26 504 (16 489)	22 564 (13 538)	18 068 (12 421)	26 019 (16 055)	22 057 (14 459)	22 421 (12 791)	20 478 (12 824)

	**Birth outcomes**							

**Preterm birth, N(%)**	131 521 (5·6)	18 503 (5·1)	58 623 (5·6)	30 989 (6·2)	4 807 (5·2)	5 890 (5·7)	5 983 (5·5)	6 726 (5·9)
**SGA, N(%)**	261 154 (11·2)	35 146 (9·8)	110 909 (10·5)	69 518 (13·8)	9 065 (9·9)	11 952 (11·5)	11 093 (10·2)	13 471 (11·8)

Time points of covariate assessment: maternal age assessed at delivery, parity is registered during the antenatal care intake, yearly household income from the year of birth of the child, and ethnicity as registered in CBS records (remains invariant across time).

Adjusted regression models show that higher odds of having a preterm birth (OR[CI]= 1·12[1·07-1·17], p<0·0001) or SGA birth (OR[CI]= 1·19[1·15-1·23], p<0·0001) were observed for women living in Stable Low SES areas, compared to women living in (the most advantaged) Stable High SES neighbourhoods (Table 3A). Moreover, women living in areas categorised as Declining to Low SES had higher odds of having a preterm birth (OR[CI]= 1·12[1·05-1·20], p=0·0014) or SGA birth (OR[CI]= 1·12[1·06-1·18], p<0·0001), as compared to women living in Stable High SES areas. Whereas odds of preterm birth were still increased for women living in an Improving to Medium SES neighbourhood (OR[CI]= 1·09[1·02-1·18], p=0·0184), this was not the case for SGA (OR[CI]= 1·04[0·98-1·10]). There were no significant differences in the odds of preterm birth or SGA between the remaining trajectories and the Stable High areas (full adjusted results in Supplementary Table 1).

**Table 3:** Odds ratios (95% CI) from multilevel logistic regression for the relationship between neighbourhood SES trajectory and birth outcomes.

A)	Full sample			
Neighbourhood SES trajectory	Preterm birth		SGA	
	Model 1	Model 2	Model 1	Model 2
Stable High	REF	REF	REF	REF
Stable Medium	1·06 (1·02-1·10)	1·04 (0·99-1·09)	1·07 (1·04-1·10)	1·03 (0·99-1·07)
Stable Low	1·20 (1·15-1·25)	1·12 (1·07-1·17)	1·37 (1·32-1·42)	1·19 (1·15-1·23)
Improving to High	0·99 (0·91-1·07)	0·98 (0·91-1·06)	0·98 (0·92-1·04)	0·97 (0·92-1·03)
Improving to Medium	1·13 (1·05-1·22)	1·09 (1·02-1·18)	1·11 (1·05-1·18)	1·04 (0·98-1·10)
Declining to Medium	1·04 (0·97-1·11)	1·03 (0·97-1·10)	1·05 (1·00-1·10)	1·03 (0·98-1·08)
Declining to Low	1·17 (1·10-1·25)	1·12 (1·05-1·20)	1·23 (1·17-1·29)	1·12 (1·06-1·18)

B)	Subsample of women who remained in the same address throughout entire exposure period			
	Preterm birth		SGA	
	Model 1	Model 2	Model 1	Model 2
Stable High	REF	REF	REF	REF
Stable Medium	1·07 (1·00-1·14)	1·05 (0·99;1·13)	1·05 (1·00;1·10)	1·02 (0·97;1·06)
Stable Low	1·26 (1·18-1·34)	1·17 (1·09;1·25)	1·34 (1·27;1·41)	1·17 (1·11;1·23)
Improving to High	1·03 (0·91-1·06)	1·02 (0·90;1·14)	0·98 (0·90;1·08)	0·98 (0·90;1·07)
Improving to Medium	1·17 (1·05-1·31)	1·12 (1·01;1·26)	1·07 (0·98;1·16)	1·00 (0·92;1·09)
Declining to Medium	1·01 (0·92-1·12)	1·00 (0·91;1·11)	1·06 (0·98;1·14)	1·04 (0·96;1·12)
Declining to Low	1·15 (1·04-1·28)	1·09 (1·01;1·18)	1·26 (1·14;1·32)	1·12 (1·04;1·20)

Model 1: Including only time-point dummy variables and time-period × neighbourhood SES trajectory interactions.

Model 2: Including Model 1 terms and adjusting for individual-level characteristics: maternal age, parity, migration background and household income.

Stable High trajectory (most advantaged) as reference category (REF).

First time-period (2003-2006) used as reference.

Number of preterm births and SGA births in each SES category are displayed in Table 2.

Part B corresponds to estimates from sensitivity analysis 3b.

Changes in the exposure-outcome relationship across time-points were assessed using interaction terms time-period × neighbourhood SES trajectory. For SGA, a downwards trend over time for the Declining to Low and Stable Low trajectories was found. For example, for the Stable Low trajectory, the odds ratios changed from 1·19 (95% 1·15-1·23) in the first period to 1·13 (95% CI 1·10 to 1·17) in the last period (Supplementary Figure 2). However, in none of the cases the interaction terms were significant. For preterm birth, the estimates remained fairly unchanged across periods (Supplementary Figure 2).

The patterns found in the main analysis remained unchanged in the sensitivity analyses where 1) models were adjusted for the time that the mother has been living in the registered neighbourhood (Supplementary Table 2), 2) we excluded births that occurred in 2006, 2010, and 2014 (Supplementary Table 3), 3) the analyses were restricted to women who had lived for at least one year (Supplementary Table 4), or the entire exposure time, in the same residential address (Table 3B and Supplementary Table 5), 4) the analyses were adjusted for maternal education (Supplementary Table 6), 5) we accounted for maternal comorbidities, and lifestyle factors (Supplementary Table 7), 6) the analyses were restricted to only spontaneous births (Supplementary Table 8), 7) observations with implausible birthweight given gestational age values were excluded (Supplementary Table 9), and 9) we fitted joint regression models (Supplementary Table 10). When restricting the analysis to women who have remained in the same address for the entire exposure period (Table 3B), the association between the Stable Low SES trajectory and preterm birth was slightly larger than in the main analysis (OR[95% CI]= 1·17 [1·09-1·25], p<0·0001). For preterm birth, the results from the main analysis remained unchanged when conducting the siblings-comparison analysis (Supplementary Table 11). For SGA, the results for the Stable Low (OR[95% CI]= 1·10 [1·04-1·16], p=0·0003) and Declining to Low (OR[95% CI]= 1·06 [1·01-1·12], p=0·0415) SES trajectories remained significant, however, the estimates were attenuated (Supplementary Table 11). Last, the E-values for the association between the Stable Low, Improving to Medium, and Declining to Low trajectories and preterm birth were 1·5, 1·4, and 1·5, respectively, indicating that the residual confounding could explain the observed association if there exists an unmeasured covariate having an association (OR) at least as large as the E-value with both the exposures and outcomes. For SGA, the E-values for the Stable Low and Declining to Low trajectories were 1·7 and 1·5.

## 4 Discussion

In this study, using a large nationwide perinatal registry linked to a comprehensive measure of neighbourhood socioeconomic status, we found a detrimental (small) association between disadvantaged neighbourhood SES trajectories and adverse birth outcomes. Women living in persistently low SES areas or areas that declined to low SES had higher odds of preterm or SGA birth, compared to women living in the most advantaged areas. Also, living in a neighbourhood whose SES shifted from low to medium was associated with higher odds of preterm birth, but not SGA. Importantly, odds of preterm or SGA birth in other areas were comparable to those seen in high SES areas.

To the best of our knowledge, this is the first study to have examined the association between changes in neighbourhood SES occurring in short (four years) periods and birth outcomes in a nationwide cohort. The findings from this study are consistent with previous evidence while adding to the literature in meaningful ways. Our finding that women in stable low SES and declining SES areas have higher odds of preterm birth and SGA births is in line with studies conducted in the US by Cubbin et al. (Texas) [6], and Magerison-Zilko et al. (California) [7]. They found that long-term neighbourhood poverty and poverty increase were associated with higher odds of preterm birth and SGA. Both studies used the changes in the percentage of persons below 100% of the federal poverty level as exposure, whereas we examined the changes in a broader measure of neighbourhood socioeconomic conditions. Moreover, our study furthers the existing literature by investigating changes occurring over shorter periods than those considered in the previous studies (20 to 40 years [6,7]). We also found that women in neighbourhoods that show gentrification (from low to medium SES) were more likely to experience preterm birth. In their study, Cubbin et al. found that living in neighbourhoods with decreasing poverty was associated with increased odds of preterm birth [6]. Additionally, Huynh et al. (New York City) found that, for disadvantaged groups, living in rapidly gentrifying neighbourhoods was associated with an increased incidence of preterm birth [11].

Previous studies have suggested lifestyle factors as mediators for the relationship between neighbourhood SES and birth outcomes [46,48]. However, in our study, we have not found any indication of mediation by these variables. There are other plausible pathways through which neighbourhood SES trajectories might impact birth outcomes. One potential explanation relates to objective neighbourhood characteristics, particularly the physical environment. Several studies have observed consistent associations between high noise and air pollution levels and adverse birth outcomes, particularly in deprived areas [52,59,60]. Moreover, inhabitants from declining and continuously deprived areas might be more exposed to deteriorating or poor built environment and housing conditions, factors that have been linked to adverse birth outcomes [60], [61], [62]. Furthermore, living in disadvantaged areas is associated with poor healthcare access and uptake, which might, in turn, affect birth outcomes [63]. This mechanism is supported by the findings from a recent European study where favourable changes in neighbourhood SES were associated with higher hypertensive pregnancies diagnosis rates [64]. Untreated hypertensive pregnancies are a well-known risk for adverse birth outcomes. A different pathway could be the psychological stress triggered by perceived neighbourhood-related factors and constant exposure to poverty-related issues [52]. For example, mothers living in declining and persistently low SES neighbourhoods might perceive lower social cohesion and safety than their counterparts living in more advantaged areas. Both aspects have been linked to adverse birth outcomes [65], [66], [67], [68]. Furthermore, neighbourhoods undergoing rapid economic improvement may also present certain stressors [11], such as rising rents and higher prices for neighbourhood resources (e.`g., stores and food outlets) [7]. Stress is hypothesised to be the main pathway for low SES neighbourhoods that are quickly improving, especially for long-term residents [11]. This might explain the differences between preterm birth and SGA in neighbourhoods improving from low to medium, as preterm birth may be more sensitive to maternal stress [69].

A unique strength of this study is its longitudinal approach towards neighbourhood social and economic conditions, which allows taking into account neighbourhoods’ dynamic nature. Using national-level routinely collected data corresponding to an extended period (2003-2017) led to over two million individual records available for analysis. By assessing several types of declining, ascending and stable neighbourhood trajectories, our results provide more precise information about the type of change that might be the most detrimental (i.e. decline to low SES). A limitation of this study is that CBS could not link some of the births, and therefore these could not be included in the analysis. However, the impact of this is likely small as the percentage of unlinked births was only around 3%. Furthermore, it cannot be ruled out that the observed association can be due to compositional effects related to the selective sorting of people into neighbourhoods [70,71]. However, previous research has found that income and ethnicity are the most important drivers of neighbourhood sorting [72], characteristics we have included in our models. We did not account for certain relevant potential confounders in the main analysis, e.g., maternal education, previous preterm birth (or SGA), hypertension, and diabetes, due to the lack of (high-quality) information. However, adding information on maternal education, hypertension and diabetes to the models in the sensitivity analyses did not change the conclusions. Furthermore, the results from the siblings-comparison analysis supported the conclusions derived from the main analysis. We computed the E-values for the statistically significant results. For example, the E-value for the Declining to Low SES trajectory was 1·5 for both preterm birth and SGA. Thus, residual confounding could explain the observed association if there exists an unmeasured covariate having an association (OR) at least as large as 1·5 with both the exposures and outcomes. Given the OR values for the known risk factors, it is not likely that an unmeasured or unknown confounder would have a substantially greater effect on adverse birth outcomes than the covariates already included.

From a public health standpoint, this study has several implications. Our findings indicate a higher risk of adverse birth outcomes for mothers living in persistently low SES neighbourhoods and areas in decline. At the same time, the odds of SGA for mothers living in improving neighbourhoods (improving to medium SES) is not significantly different from the odds for mothers in the most advantaged areas. This study suggests that longitudinal neighbourhood SES measures should also be taken into account when selecting a target population for public health interventions. Last, in agreement with previous research [34], our results indicate that even though differences in outcomes between most and least disadvantaged areas seem to be narrowing, they remain persistent [34]. Therefore, it is vital to continue public health actions to reduce this gap.

Future studies should focus on how changes in neighbourhood SES affect different strata of the population, e.g. ethnic minorities. Furthermore, future research needs to further investigate the underlying mechanisms driving the observed association, e.g., healthcare uptake and access, neighbourhood crime rates, social cohesion, air pollution, greenness, and walkability. To appropriately inform decision-makers when developing public health interventions, further research is necessary to pinpoint the causal pathways by which neighbourhood SES trajectories affect birth outcomes.

In conclusion, our results indicate that, in the Netherlands, women living in neighbourhoods with disadvantaged SES trajectories were more likely to experience adverse birth outcomes. Results from this study suggest that, longitudinal neighbourhood SES measures should also be taken into account when selecting a target population for public health interventions.

## Author Contributions

JVB and LCMB obtained funding for the study. LBO JVB and LCMB conceived the study. LBO and LCMB analysed the data. All authors were involved in interpreting the data. LBO wrote the draft paper and JVB supervised the writing. LCMB EAPS TVO and PGG provided additional input at the writing stage. All authors read and approved the final version of the manuscript.

## Data sharing

The authors are open to sharing statistical codes. Linked electronic health records require separate permission from Perined and Statistics Netherlands.

## Declaration of interests

We declare no competing interests.

## Appendix Supplementary materials

Image, application 1

## Footnotes

Supplementary material associated with this article can be found in the online version at doi:10.1016/j.lanepe.2021.100205.
