# Supplementary material for: Association of neighbourhood socioeconomic trajectories with preterm birth and small-for-gestational-age in the Netherlands: a nationwide population-based study
Source: Lancet Reg Health Eur. 2021 Aug 24;10:100205. doi: 10.1016/j.lanepe.2021.100205 (PMC8589710; doi:10.1016/j.lanepe.2021.100205)

**SUPPLEMENTARY MATERIAL**

**Supplementary table 1. Full results for main adjusted models (N= 2 334 036).**

| **Variable** | **Preterm birth** | **SGA** |
| --- | --- | --- |
| Stable medium | 1.04(0.99;1.09) | 1.03(0.99;1.07) |
| Stable low | 1.12(1.07;1.17) | 1.19(1.15;1.23) |
| Improving to high | 0.98(0.91;1.06) | 0.97(0.92;1.03) |
| Improving to medium | 1.09(1.02;1.18) | 1.04(0.98;1.10) |
| Declining to medium | 1.03(0.97;1.10) | 1.03(0.98;1.08) |
| Declining to low | 1.12(1.05;1.20) | 1.12(1.06;1.18) |
| Cohort 2 | 0.98(0.94;1.03) | 0.91(0.88;0.94) |
| Cohort 3 | 0.97(0.93;1.02) | 0.92(0.89;0.95) |
| Cohort 4 | 0.92(0.88;0.96) | 0.93(0.90;0.96) |
| Maternal age (20-34) | 1.01(0.97;1.06) | 0.97(0.94;1.00) |
| Maternal age (>35) | 1.21(1.16;1.27) | 1.15(1.11;1.19) |
| Multiparous | 0.51(0.51;0.52) | 0.46(0.45;0.46) |
| Moroccan | 0.81(0.78;0.83) | 0.92(0.90;0.94) |
| Turkish | 0.91(0.88;0.94) | 1.08(1.05;1.11) |
| Suriname | 1.47(1.42;1.52) | 2.42(2.37;2.48) |
| Antillean | 1.33(1.27;1.39) | 1.44(1.39;1.49) |
| Other non-western | 0.93(0.90;0.95) | 1.33(1.30;1.35) |
| Other western | 0.90(0.87;0.92) | 1.06(1.04;1.07) |
| Migrant first generation | 0.94(0.91;0.96) | 0.92(0.90;0.94) |
| Migrant second generation | 1.06(1.04;1.09) | 1.08(1.06;1.10) |
| Household income Q2 | 0.87(0.85;0.89) | 0.84(0.83;0.86) |
| Household income Q3 | 0.84(0.82;0.85) | 0.73(0.72;0.74) |
| Household income Q4 | 0.81(0.79;0.82) | 0.65(0.64;0.65) |
| Household income Q5 (highest) | 0.78(0.76;0.79) | 0.60(0.60;0.61) |
| Stable medium:Cohort 2 | 1.04(0.99;1.09) | 1.05(1.01;1.09) |
| Stable low:Cohort 2 | 1.02(0.96;1.08) | 1.01(0.97;1.05) |
| Improving to high:Cohort 2 | 1.09(0.97;1.21) | 1.04(0.96;1.14) |
| Improving to medium:Cohort 2 | 0.98(0.89;1.09) | 1.04(0.96;1.13) |
| Declining to medium:Cohort 2 | 1.03(0.94;1.11) | 0.99(0.93;1.06) |
| Declining to low:Cohort 2 | 0.97(0.89;1.05) | 0.98(0.91;1.04) |
| Stable medium:Cohort 3 | 1.03(0.98;1.08) | 1.00(0.96;1.04) |
| Stable low:Cohort 3 | 1.01(0.95;1.06) | 0.96(0.92;1.00) |
| Improving to high:Cohort 3 | 1.04(0.94;1.14) | 1.02(0.95;1.10) |
| Improving to medium:Cohort 3 | 0.94(0.86;1.03) | 1.04(0.98;1.12) |
| Declining to medium:Cohort 3 | 1.05(0.95;1.16) | 0.95(0.88;1.03) |
| Declining to low:Cohort 3 | 0.95(0.87;1.04) | 0.90(0.84;0.96) |
| Stable medium:Cohort 4 | 1.03(0.98;1.08) | 0.98(0.94;1.02) |
| Stable low:Cohort 4 | 1.02(0.96;1.07) | 0.95(0.91;0.99) |
| Improving to high:Cohort 4 | 1.01(0.90;1.13) | 1.05(0.96;1.14) |
| Improving to medium:Cohort 4 | 0.99(0.89;1.10) | 0.95(0.88;1.04) |
| Declining to medium:Cohort 4 | 1.00(0.91;1.11) | 0.97(0.90;1.04) |
| Declining to low:Cohort 4 | 0.98(0.90;1.07) | 0.94(0.88;1.01) |
| Models adjusted for maternal age, parity, migration background (ethnicity and generation) and household income.  For categorical variables, omitted categories are reference categories. | | |

**Supplementary table 2. Sensitivity analysis 1: Models adjusted for individual-level characteristics and duration of residence in the neighbourhood (N= 2 334 036).**

| **Variable** | **Preterm birth** | **SGA** |
| --- | --- | --- |
| Stable medium | 1.04(0.99;1.09) | 1.03(0.99;1.07) |
| Stable low | 1.12(1.08;1.17) | 1.19(1.15;1.23) |
| Improving to high | 0.98(0.91;1.06) | 0.97(0.92;1.03) |
| Improving to medium | 1.09(1.02;1.18) | 1.04(0.98;1.10) |
| Declining to medium | 1.03(0.97;1.10) | 1.03(0.98;1.08) |
| Declining to low | 1.12(1.05;1.20) | 1.12(1.06;1.17) |
| Cohort 2 | 0.99(0.94;1.03) | 0.91(0.88;0.94) |
| Cohort 3 | 0.97(0.93;1.02) | 0.92(0.89;0.95) |
| Cohort 4 | 0.93(0.89;0.97) | 0.92(0.89;0.95) |
| Maternal age (20-34) | 1.01(0.97;1.06) | 0.98(0.95;1.01) |
| Maternal age (>35) | 1.21(1.15;1.26) | 1.16(1.12;1.19) |
| Multiparous | 0.51(0.51;0.52) | 0.46(0.45;0.46) |
| Moroccan | 0.81(0.78;0.84) | 0.92(0.90;0.94) |
| Turkish | 0.91(0.88;0.94) | 1.08(1.06;1.11) |
| Suriname | 1.47(1.42;1.52) | 2.42(2.37;2.48) |
| Antillean | 1.33(1.27;1.39) | 1.44(1.39;1.48) |
| Other non-western | 0.93(0.91;0.95) | 1.33(1.30;1.35) |
| Other western | 0.89(0.87;0.92) | 1.05(1.04;1.07) |
| Migrant first generation | 0.93(0.91;0.96) | 0.92(0.90;0.94) |
| Migrant second generation | 1.07(1.04;1.09) | 1.08(1.06;1.10) |
| Household income Q2 | 0.87(0.85;0.89) | 0.85(0.83;0.86) |
| Household income Q3 | 0.84(0.82;0.85) | 0.73(0.72;0.74) |
| Household income Q4 | 0.81(0.79;0.83) | 0.65(0.64;0.66) |
| Household income Q5 (highest) | 0.78(0.76;0.79) | 0.61(0.60;0.62) |
| Time in address | 1.00(1.00;1.00) | 1.00(1.00;1.00) |
| Stable medium:Cohort 2 | 1.03(0.98;1.09) | 1.05(1.01;1.09) |
| Stable low:Cohort 2 | 1.02(0.96;1.07) | 1.01(0.97;1.05) |
| Improving to high:Cohort 2 | 1.08(0.97;1.21) | 1.04(0.96;1.14) |
| Improving to medium:Cohort 2 | 0.98(0.89;1.09) | 1.04(0.96;1.13) |
| Declining to medium:Cohort 2 | 1.03(0.94;1.11) | 1.00(0.93;1.06) |
| Declining to low:Cohort 2 | 0.97(0.88;1.05) | 0.98(0.92;1.05) |
| Stable medium:Cohort 3 | 1.03(0.98;1.08) | 1.00(0.96;1.04) |
| Stable low:Cohort 3 | 1.00(0.95;1.06) | 0.97(0.93;1.01) |
| Improving to high:Cohort 3 | 1.04(0.94;1.14) | 1.02(0.95;1.10) |
| Improving to medium:Cohort 3 | 0.94(0.86;1.03) | 1.04(0.98;1.12) |
| Declining to medium:Cohort 3 | 1.05(0.95;1.16) | 0.95(0.88;1.03) |
| Declining to low:Cohort 3 | 0.95(0.87;1.04) | 0.90(0.84;0.96) |
| Stable medium:Cohort 4 | 1.03(0.98;1.08) | 0.98(0.94;1.02) |
| Stable low:Cohort 4 | 1.01(0.96;1.07) | 0.95(0.92;0.99) |
| Improving to high:Cohort 4 | 1.00(0.89;1.13) | 1.05(0.96;1.14) |
| Improving to medium:Cohort 4 | 0.99(0.89;1.10) | 0.95(0.88;1.04) |
| Declining to medium:Cohort 4 | 1.00(0.91;1.11) | 0.97(0.90;1.04) |
| Declining to low:Cohort 4 | 0.98(0.90;1.07) | 0.95(0.89;1.01) |
| Odds ratios (95% CI).  Models adjusted for maternal age, parity, migration background (ethnicity and generation) and household income and duration of residence in the neighbourhood.  For categorical variables, omitted categories are reference categories. | | |

**Supplementary table 3. Models excluding observations from years 2006, 2010 and 2014 from the analysis (N= 1 860 958).**

| **Variable** | **Preterm birth** | **SGA** |
| --- | --- | --- |
| Stable medium | 1.05(0.99;1.10) | 1.04(0.99;1.08) |
| Stable low | 1.12(1.08;1.17) | 1.20(1.16;1.25) |
| Improving to high | 0.98(0.91;1.06) | 0.97(0.91;1.03) |
| Improving to medium | 1.09(1.01;1.18) | 1.05(0.99;1.11) |
| Declining to medium | 1.03(0.97;1.10) | 1.04(0.98;1.09) |
| Declining to low | 1.12(1.05;1.19) | 1.12(1.07;1.18) |
| Cohort 2 | 0.98(0.94;1.03) | 0.90(0.87;0.93) |
| Cohort 3 | 0.97(0.93;1.02) | 0.95(0.91;0.98) |
| Cohort 4 | 0.92(0.88;0.97) | 0.93(0.90;0.97) |
| Maternal age (20-34) | 1.00(0.95;1.05) | 0.98(0.94;1.01) |
| Maternal age (>35) | 1.19(1.13;1.25) | 1.15(1.11;1.20) |
| Multiparous | 0.51(0.50;0.51) | 0.46(0.45;0.46) |
| Moroccan | 0.81(0.79;0.85) | 0.92(0.89;0.94) |
| Turkish | 0.91(0.88;0.95) | 1.09(1.06;1.12) |
| Suriname | 1.47(1.42;1.53) | 2.43(2.37;2.49) |
| Antillean | 1.37(1.30;1.44) | 1.45(1.40;1.50) |
| Other non-western | 0.94(0.91;0.96) | 1.33(1.31;1.36) |
| Other western | 0.90(0.88;0.92) | 1.06(1.04;1.08) |
| Migrant first generation | 0.94(0.91;0.96) | 0.92(0.90;0.94) |
| Migrant second generation | 1.07(1.04;1.09) | 1.08(1.06;1.10) |
| Household income Q2 | 0.87(0.85;0.89) | 0.85(0.83;0.86) |
| Household income Q3 | 0.84(0.82;0.86) | 0.73(0.72;0.74) |
| Household income Q4 | 0.81(0.80;0.83) | 0.64(0.63;0.65) |
| Household income Q5 (highest) | 0.78(0.76;0.80) | 0.60(0.59;0.61) |
| Stable medium:Cohort 2 | 1.04(0.98;1.10) | 1.04(1.00;1.08) |
| Stable low:Cohort 2 | 1.00 (0.94;1.06) | 1.00 (0.96;1.05) |
| Improving to high:Cohort 2 | 1.07(0.95;1.20) | 1.07(0.97;1.17) |
| Improving to medium:Cohort 2 | 0.99(0.89;1.11) | 1.04(0.95;1.13) |
| Declining to medium:Cohort 2 | 1.01(0.93;1.10) | 0.98(0.91;1.05) |
| Declining to low:Cohort 2 | 0.98(0.89;1.08) | 0.97(0.91;1.04) |
| Stable medium:Cohort 3 | 1.02(0.96;1.07) | 0.98(0.94;1.02) |
| Stable low:Cohort 3 | 0.99(0.93;1.05) | 0.95(0.91;1.00) |
| Improving to high:Cohort 3 | 1.03(0.93;1.14) | 1.02(0.94;1.10) |
| Improving to medium:Cohort 3 | 0.94(0.86;1.04) | 1.03(0.96;1.10) |
| Declining to medium:Cohort 3 | 1.06(0.95;1.19) | 0.95(0.87;1.04) |
| Declining to low:Cohort 3 | 0.92(0.83;1.01) | 0.88(0.81;0.94) |
| Stable medium:Cohort 4 | 1.02(0.96;1.07) | 0.98(0.94;1.02) |
| Stable low:Cohort 4 | 1.00 (0.95;1.07) | 0.95(0.91;0.99) |
| Improving to high:Cohort 4 | 1.00 (0.88;1.14) | 1.08(0.98;1.19) |
| Improving to medium:Cohort 4 | 0.96(0.85;1.08) | 0.93(0.85;1.02) |
| Declining to medium:Cohort 4 | 0.99(0.89;1.10) | 0.97(0.89;1.05) |
| Declining to low:Cohort 4 | 1.01(0.92;1.11) | 0.94(0.88;1.01) |
| Odds ratios (95% CI).  Models adjusted for maternal age, parity, migration background (ethnicity and generation) and household income.  For categorical variables, omitted categories are reference categories. | | |

**Supplementary table 4. Sensitivity analysis 3a: Analysis restricted to women that have been living for at least 1 year in the same address at the time of delivery(N=2 274 449).**

| **Variable** | **Preterm birth** | **SGA** |
| --- | --- | --- |
| Stable medium | 1.04(0.99;1.09) | 1.03(0.99;1.08) |
| Stable low | 1.11(1.07;1.16) | 1.19(1.15;1.24) |
| Improving to high | 0.97(0.89;1.05) | 0.99(0.93;1.05) |
| Improving to medium | 1.10(1.02;1.18) | 1.04(0.99;1.11) |
| Declining to medium | 1.03(0.96;1.10) | 1.03(0.98;1.08) |
| Declining to low | 1.11(1.04;1.19) | 1.13(1.07;1.18) |
| Cohort 2 | 0.98(0.94;1.03) | 0.91(0.88;0.94) |
| Cohort 3 | 0.97(0.93;1.01) | 0.92(0.89;0.96) |
| Cohort 4 | 0.92(0.88;0.96) | 0.93(0.90;0.96) |
| Maternal age (20-34) | 1.03(0.98;1.08) | 0.97(0.94;1.00) |
| Maternal age (>35) | 1.23(1.17;1.30) | 1.15(1.11;1.19) |
| Multiparous | 0.51(0.50;0.52) | 0.46(0.45;0.46) |
| Moroccan | 0.82(0.79;0.84) | 0.93(0.91;0.95) |
| Turkish | 0.92(0.89;0.95) | 1.09(1.07;1.12) |
| Suriname | 1.47(1.42;1.52) | 2.45(2.39;2.50) |
| Antillean | 1.33(1.27;1.40) | 1.44(1.39;1.49) |
| Other non-western | 0.93(0.91;0.96) | 1.34(1.31;1.36) |
| Other western | 0.90(0.88;0.92) | 1.06(1.04;1.08) |
| Migrant first generation | 0.94(0.91;0.96) | 0.92(0.90;0.94) |
| Migrant second generation | 1.06(1.04;1.09) | 1.08(1.06;1.10) |
| Household income Q2 | 0.87(0.86;0.89) | 0.85(0.84;0.86) |
| Household income Q3 | 0.84(0.82;0.86) | 0.74(0.73;0.75) |
| Household income Q4 | 0.81(0.80;0.83) | 0.65(0.64;0.66) |
| Household income Q5 (highest) | 0.78(0.76;0.80) | 0.61(0.60;0.62) |
| Stable medium:Cohort 2 | 1.04(0.99;1.09) | 1.05(1.01;1.09) |
| Stable low:Cohort 2 | 1.02(0.96;1.08) | 1.01(0.97;1.05) |
| Improving to high:Cohort 2 | 1.11(0.99;1.23) | 1.04(0.95;1.13) |
| Improving to medium:Cohort 2 | 0.97(0.88;1.08) | 1.05(0.97;1.14) |
| Declining to medium:Cohort 2 | 1.03(0.94;1.12) | 0.99(0.93;1.06) |
| Declining to low:Cohort 2 | 0.97(0.89;1.06) | 0.97(0.91;1.04) |
| Stable medium:Cohort 3 | 1.03(0.98;1.09) | 0.99(0.96;1.03) |
| Stable low:Cohort 3 | 1.01(0.96;1.07) | 0.96(0.92;1.01) |
| Improving to high:Cohort 3 | 1.05(0.95;1.16) | 1.01(0.94;1.09) |
| Improving to medium:Cohort 3 | 0.94(0.86;1.03) | 1.04(0.97;1.12) |
| Declining to medium:Cohort 3 | 1.06(0.96;1.18) | 0.95(0.88;1.03) |
| Declining to low:Cohort 3 | 0.96(0.88;1.06) | 0.89(0.83;0.96) |
| Stable medium:Cohort 4 | 1.03(0.98;1.08) | 0.98(0.94;1.01) |
| Stable low:Cohort 4 | 1.02(0.97;1.08) | 0.95(0.91;0.99) |
| Improving to high:Cohort 4 | 1.03(0.91;1.16) | 1.03(0.95;1.13) |
| Improving to medium:Cohort 4 | 0.98(0.88;1.09) | 0.94(0.87;1.03) |
| Declining to medium:Cohort 4 | 1.02(0.92;1.13) | 0.97(0.90;1.05) |
| Declining to low:Cohort 4 | 0.99(0.91;1.08) | 0.95(0.89;1.01) |
| Odds ratios (95% CI).  Models adjusted for maternal age, parity, migration background (ethnicity and generation) and household income.  For categorical variables, omitted categories are reference categories. | | |

**Supplementary table 5. Sensitivity analysis 3b: Analysis restricted to women living in the same address throughout the entire 4-years period corresponding to the assigned exposure (neighbourhood SES trajectory) (N= 1 164 759).**

| **Variable** | **Preterm birth** | **SGA** |
| --- | --- | --- |
| Stable medium | 1.05(0.99;1.13) | 1.02(0.97;1.06) |
| Stable low | 1.17(1.09;1.25) | 1.17(1.11;1.23) |
| Improving to high | 1.02(0.90;1.14) | 0.98(0.90;1.07) |
| Improving to medium | 1.12(1.01;1.26) | 1.00(0.92;1.09) |
| Declining to medium | 1.00(0.91;1.11) | 1.04(0.96;1.12) |
| Declining to low | 1.09(1.01;1.18) | 1.12(1.04;1.20) |
| Cohort 2 | 1.00(0.93;1.06) | 0.90(0.86;0.95) |
| Cohort 3 | 0.99(0.93;1.06) | 0.87(0.83;0.91) |
| Cohort 4 | 0.96(0.90;1.02) | 0.89(0.85;0.93) |
| Maternal age (20-34) | 1.06(0.98;1.14) | 0.96(0.90;1.01) |
| Maternal age (>35) | 1.30(1.20;1.41) | 1.14(1.07;1.20) |
| Multiparous | 0.46(0.45;0.47) | 0.43(0.42;0.43) |
| Moroccan | 0.89(0.85;0.94) | 0.95(0.91;0.98) |
| Turkish | 1.07(1.02;1.12) | 1.16(1.12;1.20) |
| Suriname | 1.63(1.55;1.71) | 2.60(2.51;2.69) |
| Antillean | 1.45(1.35;1.55) | 1.49(1.41;1.57) |
| Other non-western | 1.10(1.05;1.14) | 1.34(1.30;1.38) |
| Other western | 0.98(0.95;1.02) | 1.09(1.06;1.12) |
| Migrant first generation | 0.99(0.93;1.03) | 0.92(0.90;0.94) |
| Migrant second generation | 1.01(0.97;1.04) | 1.07(1.05;1.10) |
| Household income Q2 | 0.87(0.85;0.90) | 0.85(0.83;0.87) |
| Household income Q3 | 0.84(0.81;0.86) | 0.73(0.72;0.75) |
| Household income Q4 | 0.80(0.78;0.82) | 0.66(0.64;0.67) |
| Household income Q5 (highest) | 0.77(0.74;0.79) | 0.62(0.60;0.63) |
| Stable medium:Cohort 2 | 1.03(0.96;1.11) | 1.06(1.00;1.12) |
| Stable low:Cohort 2 | 0.97(0.90;1.06) | 1.03(0.97;1.09) |
| Improving to high:Cohort 2 | 1.06(0.90;1.24) | 1.03(0.91;1.16) |
| Improving to medium:Cohort 2 | 0.94(0.81;1.09) | 1.05(0.94;1.18) |
| Declining to medium:Cohort 2 | 1.08(0.96;1.23) | 1.00(0.91;1.10) |
| Declining to low:Cohort 2 | 0.99(0.86;1.12) | 0.99(0.90;1.09) |
| Stable medium:Cohort 3 | 1.02(0.94;1.09) | 1.03(0.97;1.09) |
| Stable low:Cohort 3 | 0.95(0.87;1.03) | 1.01(0.95;1.07) |
| Improving to high:Cohort 3 | 0.99(0.86;1.14) | 1.02(0.92;1.14) |
| Improving to medium:Cohort 3 | 0.90(0.79;1.02) | 1.14(1.03;1.26) |
| Declining to medium:Cohort 3 | 0.99(0.85;1.15) | 0.98(0.88;1.11) |
| Declining to low:Cohort 3 | 0.96(0.84;1.09) | 0.92(0.83;1.02) |
| Stable medium:Cohort 4 | 0.98(0.91;1.06) | 1.00(0.95;1.06) |
| Stable low:Cohort 4 | 0.96(0.88;1.04) | 0.98(0.92;1.05) |
| Improving to high:Cohort 4 | 0.94(0.79;1.11) | 1.05(0.92;1.19) |
| Improving to medium:Cohort 4 | 0.93(0.79;1.09) | 0.99(0.87;1.12) |
| Declining to medium:Cohort 4 | 1.04(0.90;1.20) | 0.98(0.88;1.09) |
| Declining to low:Cohort 4 | 1.01(0.89;1.16) | 0.98(0.89;1.08) |
| Odds ratios (95% CI).  Models adjusted for maternal age, parity, migration background (ethnicity and generation) and household income and.  For categorical variables, omitted categories are reference categories. | | |

**Supplementary table 6. Sensitivity analysis 4: Additionally adjusting for maternal educational level (N= 2 334 036).**

| **Neighbourhood trajectory** | **Preterm birth** | **SGA** |
| --- | --- | --- |
| Stable medium | 1.03(0.99;1.07) | 1.02(0.99;1.05) |
| Stable low | 1.11(1.06;1.16) | 1.18(1.14;1.22) |
| Improving to high | 0.98(0.90;1.06) | 0.97(0.91;1.03) |
| Improving to medium | 1.08(1.01;1.17) | 1.03(0.97;1.09) |
| Declining to medium | 1.02(0.96;1.09) | 1.02(0.97;1.07) |
| Declining to low | 1.10(1.03;1.18) | 1.11(1.05;1.16) |
| Cohort 2 | 0.99(0.95;1.04) | 0.92(0.89;0.95) |
| Cohort 3 | 0.98(0.94;1.02) | 0.94(0.91;0.97) |
| Cohort 4 | 0.93(0.89;0.97) | 0.95(0.92;0.99) |
| Maternal age (20-34) | 1.13(1.08;1.18) | 1.11(1.08;1.15) |
| Maternal age (>35) | 1.36(1.30;1.43) | 1.32(1.28;1.37) |
| Multiparous | 0.52(0.51;0.52) | 0.46(0.45;0.46) |
| Moroccan | 0.74(0.72;0.76) | 0.83(0.81;0.85) |
| Turkish | 0.83(0.80;0.86) | 0.97(0.95;1.00) |
| Suriname | 1.39(1.35;1.44) | 2.29(2.24;2.34) |
| Antillean | 1.27(1.22;1.33) | 1.37(1.33;1.42) |
| Other non-western | 0.87(0.85;0.89) | 1.23(1.21;1.25) |
| Other western | 0.86(0.84;0.88) | 1.01(0.99;1.02) |
| Migrant first generation | 0.89(0.87;0.92) | 0.86(0.84;0.88) |
| Migrant second generation | 1.11(1.08;1.13) | 1.14(1.12;1.16) |
| Household income Q2 | 0.90(0.88;0.92) | 0.88(0.87;0.89) |
| Household income Q3 | 0.89(0.87;0.91) | 0.78(0.77;0.79) |
| Household income Q4 | 0.90(0.88;0.91) | 0.72(0.71;0.73) |
| Household income Q5 (highest) | 0.90(0.88;0.92) | 0.71(0.70;0.73) |
| Stable medium:Cohort 2 | 1.03(0.98;1.08) | 1.04(1.00;1.08) |
| Stable low:Cohort 2 | 1.01(0.95;1.06) | 1.00(0.96;1.04) |
| Improving to high:Cohort 2 | 1.08(0.97;1.20) | 1.04(0.95;1.13) |
| Improving to medium:Cohort 2 | 0.97(0.88;1.08) | 1.03(0.95;1.12) |
| Declining to medium:Cohort 2 | 1.03(0.95;1.12) | 1.00(0.93;1.06) |
| Declining to low:Cohort 2 | 0.97(0.89;1.05) | 0.97(0.91;1.04) |
| Stable medium:Cohort 3 | 1.01(0.96;1.07) | 0.98(0.95;1.02) |
| Stable low:Cohort 3 | 0.99(0.94;1.05) | 0.95(0.91;0.99) |
| Improving to high:Cohort 3 | 1.03(0.94;1.14) | 1.02(0.94;1.09) |
| Improving to medium:Cohort 3 | 0.94(0.86;1.02) | 1.03(0.97;1.10) |
| Declining to medium:Cohort 3 | 1.05(0.94;1.16) | 0.95(0.88;1.03) |
| Declining to low:Cohort 3 | 0.94(0.86;1.03) | 0.88(0.82;0.95) |
| Stable medium:Cohort 4 | 1.01(0.97;1.07) | 0.97(0.93;1.00) |
| Stable low:Cohort 4 | 1.01(0.95;1.06) | 0.94(0.90;0.98) |
| Improving to high:Cohort 4 | 1.00(0.89;1.12) | 1.04(0.95;1.13) |
| Improving to medium:Cohort 4 | 0.98(0.88;1.09) | 0.94(0.87;1.02) |
| Declining to medium:Cohort 4 | 1.00(0.91;1.10) | 0.96(0.89;1.04) |
| Declining to low:Cohort 4 | 0.97(0.89;1.06) | 0.93(0.87;0.99) |
| Mother’s education (medium) | 1.27(1.25;1.29) | 1.31(1.29;1.32) |
| Mother’s education (low) | 1.57(1.53;1.61) | 1.70(1.67;1.72) |
| Mother’s education (missing) | 1.22(1.20;1.24) | 1.28(1.26;1.30) |
| Odds ratios (95% CI).  Models adjusted for maternal age, parity, migration background (ethnicity and generation), household income, and morther’s education.  For categorical variables, omitted categories are reference categories.  Mother’s education categories (percentage of total N): High (33.46%),Medium (38.12%), Low (8.81%), and Missing(19.61%). | | |

**Supplementary table 7. Sensitivity analysis 5: Additionally adjusting for maternal comorbidities (pre-existing diabetes and hypertension), and unhealthy life-style factors (smoking, alcohol consumption, drug use) (N= 2 334 036).**

| **Variable** | **Preterm birth** | | **SGA** | |
| --- | --- | --- | --- | --- |
|  | Model A | Model B | Model A | Model B |
| Stable medium | 1.04(0.99;1.09) | 1.04(0.99;1.09) | 1.03(0.99;1.06) | 1.03(0.99;1.07) |
| Stable low | 1.12(1.07;1.17) | 1.12(1.07;1.17) | 1.19(1.15;1.23) | 1.19(1.15;1.23) |
| Improving to high | 0.98(0.91;1.06) | 0.98(0.91;1.06) | 0.97(0.92;1.03) | 0.97(0.92;1.03) |
| Improving to medium | 1.09(1.02;1.18) | 1.09(1.02;1.18) | 1.04(0.98;1.10) | 1.04(0.98;1.10) |
| Declining to medium | 1.03(0.97;1.10) | 1.03(0.97;1.10) | 1.03(0.98;1.08) | 1.03(0.98;1.08) |
| Declining to low | 1.12(1.05;1.20) | 1.12(1.05;1.20) | 1.12(1.07;1.18) | 1.12(1.06;1.17) |
| Cohort 2 | 0.98(0.94;1.03) | 0.98(0.94;1.03) | 0.91(0.88;0.94) | 0.91(0.88;0.94) |
| Cohort 3 | 0.97(0.93;1.01) | 0.97(0.93;1.01) | 0.92(0.89;0.95) | 0.92(0.89;0.95) |
| Cohort 4 | 0.92(0.88;0.96) | 0.92(0.88;0.96) | 0.93(0.90;0.96) | 0.93(0.90;0.96) |
| Maternal age (20-34) | 1.01(0.96;1.05) | 1.01(0.97;1.06) | 0.97(0.94;1.00) | 0.98(0.95;1.01) |
| Maternal age (>35) | 1.20(1.15;1.26) | 1.20(1.15;1.26) | 1.15(1.11;1.18) | 1.15(1.12;1.19) |
| Multiparous | 0.51(0.51;0.52) | 0.51(0.51;0.52) | 0.46(0.45;0.46) | 0.46(0.45;0.46) |
| Moroccan | 0.81(0.78;0.83) | 0.81(0.78;0.83) | 0.92(0.90;0.94) | 0.93(0.91;0.95) |
| Turkish | 0.91(0.88;0.94) | 0.91(0.88;0.94) | 1.08(1.06;1.11) | 1.09(1.06;1.11) |
| Suriname | 1.46(1.41;1.51) | 1.46(1.41;1.51) | 2.43(2.37;2.48) | 2.44(2.38;2.49) |
| Antillean | 1.32(1.26;1.39) | 1.33(1.27;1.39) | 1.44(1.39;1.49) | 1.45(1.40;1.50) |
| Other non-western | 0.93(0.90;0.95) | 0.93(0.91;0.95) | 1.33(1.31;1.35) | 1.34(1.32;1.37) |
| Other western | 0.90(0.88;0.92) | 0.90(0.88;0.92) | 1.06(1.04;1.07) | 1.06(1.04;1.08) |
| Migrant first generation | 0.94(0.91;0.96) | 0.94(0.91;0.96) | 0.92(0.90;0.94) | 0.92(0.90;0.94) |
| Migrant second generation | 1.06(1.04;1.09) | 1.06(1.04;1.09) | 1.08(1.06;1.10) | 1.08(1.06;1.10) |
| Household income Q2 | 0.87(0.85;0.89) | 0.87(0.86;0.89) | 0.84(0.83;0.86) | 0.85(0.84;0.86) |
| Household income Q3 | 0.84(0.82;0.85) | 0.84(0.82;0.85) | 0.73(0.72;0.74) | 0.74(0.73;0.75) |
| Household income Q4 | 0.81(0.79;0.82) | 0.81(0.80;0.83) | 0.65(0.64;0.65) | 0.65(0.64;0.66) |
| Household income Q5 (highest) | 0.78(0.76;0.79) | 0.78(0.76;0.80) | 0.60(0.60;0.61) | 0.61(0.60;0.62) |
| Hypertension | 2.25(2.08;2.42) | 2.25(2.09;2.43) | 1.73(1.62;1.84) | 1.73(1.63;1.85) |
| Diabetes | 3.00(2.73;3.30) | 3.00(2.73;3.30) | 3.10(2.75;3.28) | 0.42(0.36;0.48) |
| Stable medium:Cohort 2 | 1.04(0.98;1.09) | 1.04(0.98;1.09) | 1.05(1.01;1.09) | 1.05(1.01;1.09) |
| Stable low:Cohort 2 | 1.02(0.96;1.08) | 1.02(0.96;1.08) | 1.01(0.97;1.05) | 1.01(0.97;1.05) |
| Improving to high:Cohort 2 | 1.08(0.97;1.21) | 1.08(0.97;1.21) | 1.04(0.96;1.14) | 1.04(0.96;1.14) |
| Improving to medium:Cohort 2 | 0.98(0.89;1.09) | 0.98(0.89;1.09) | 1.04(0.96;1.13) | 1.04(0.96;1.13) |
| Declining to medium:Cohort 2 | 1.03(0.94;1.11) | 1.03(0.94;1.11) | 0.99(0.93;1.06) | 1.00(0.93;1.06) |
| Declining to low:Cohort 2 | 0.97(0.89;1.05) | 0.97(0.89;1.05) | 0.98(0.91;1.04) | 0.98(0.92;1.04) |
| Stable medium:Cohort 3 | 1.03(0.98;1.08) | 1.03(0.98;1.08) | 1.00(0.96;1.04) | 1.00(0.96;1.04) |
| Stable low:Cohort 3 | 1.00(0.95;1.06) | 1.00(0.95;1.06) | 0.96(0.92;1.00) | 0.96(0.93;1.01) |
| Improving to high:Cohort 3 | 1.04(0.94;1.14) | 1.03(0.94;1.14) | 1.02(0.95;1.10) | 1.02(0.95;1.10) |
| Improving to medium:Cohort 3 | 0.94(0.86;1.03) | 0.94(0.86;1.03) | 1.04(0.97;1.11) | 1.04(0.97;1.11) |
| Declining to medium:Cohort 3 | 1.05(0.95;1.16) | 1.05(0.95;1.16) | 0.95(0.88;1.03) | 0.95(0.88;1.03) |
| Declining to low:Cohort 3 | 0.95(0.87;1.04) | 0.95(0.87;1.04) | 0.90(0.84;0.96) | 0.90(0.84;0.96) |
| Stable medium:Cohort 4 | 1.03(0.98;1.08) | 1.03(0.98;1.08) | 0.98(0.94;1.02) | 0.97(0.94;1.01) |
| Stable low:Cohort 4 | 1.01(0.96;1.07) | 1.01(0.96;1.07) | 0.95(0.91;0.99) | 0.94(0.90;0.98) |
| Improving to high:Cohort 4 | 1.01(0.90;1.13) | 1.01(0.89;1.13) | 1.05(0.96;1.14) | 1.04(0.96;1.14) |
| Improving to medium:Cohort 4 | 0.99(0.89;1.10) | 0.99(0.89;1.10) | 0.95(0.88;1.04) | 0.95(0.87;1.03) |
| Declining to medium:Cohort 4 | 1.00(0.91;1.10) | 1.00(0.91;1.10) | 0.97(0.90;1.04) | 0.97(0.90;1.04) |
| Declining to low:Cohort 4 | 0.98(0.90;1.07) | 0.98(0.90;1.07) | 0.94(0.88;1.01) | 0.94(0.88;1.00) |
| Drug use |  | 1.71(1.42;2.05) |  | 2.62(2.31;2.97) |
| Alcohol consumption |  | 1.70(0.90;3.22) |  | 2.90(1.86;4.51) |
| Smoking |  | 1.24(1.15;1.34) |  | 2.31(2.21;2.43) |
| Odds ratios (95% CI).  Models A adjusted for maternal age, parity, migration background (ethnicity and generation), pre-existent hypertension and diabetes, and household income.  Model B additionally adjusted for smoking, alcohol consumption, drug abuse.  For categorical variables, omitted categories are reference categories. | | | | |

**Supplementary table 8. Sensitivity analysis 6: Restricted the analysis to only spontaneous births (N= 1 757 999).**

| **Variable** | **Preterm birth** | **SGA** |
| --- | --- | --- |
| Stable medium | 1.04(0.99;1.09) | 1.04(0.99;1.09) |
| Stable low | 1.12(1.07;1.17) | 1.21(1.16;1.25) |
| Improving to high | 0.99(0.91;1.07) | 0.99(0.93;1.06) |
| Improving to medium | 1.09(1.01;1.17) | 1.06(0.99;1.13) |
| Declining to medium | 1.02(0.95;1.09) | 1.02(0.96;1.08) |
| Declining to low | 1.12(1.05;1.20) | 1.13(1.07;1.20) |
| Cohort 2 | 0.98(0.94;1.02) | 0.93(0.89;0.96) |
| Cohort 3 | 0.97(0.93;1.01) | 0.93(0.90;0.97) |
| Cohort 4 | 0.92(0.88;0.96) | 0.93(0.9;0.97) |
| Maternal age (20-34) | 1.02(0.97;1.07) | 0.98(0.94;1.01) |
| Maternal age (>35) | 1.21(1.16;1.27) | 1.16(1.11;1.20) |
| Multiparous | 0.52(0.51;0.52) | 0.47(0.47;0.48) |
| Moroccan | 0.79(0.76;0.82) | 0.98(0.96;1.01) |
| Turkish | 0.89(0.86;0.92) | 1.09(1.06;1.12) |
| Suriname | 1.42(1.37;1.47) | 2.51(2.45;2.58) |
| Antillean | 1.26(1.20;1.32) | 1.46(1.40;1.52) |
| Other non-western | 0.91(0.88;0.93) | 1.39(1.36;1.42) |
| Other western | 0.89(0.87;0.91) | 1.09(1.07;1.11) |
| Migrant first generation | 0.94(0.91;0.96) | 0.92(0.90;0.94) |
| Migrant second generation | 1.07(1.04;1.10) | 1.08(1.06;1.10) |
| Household income Q2 | 0.87(0.86;0.89) | 0.85(0.84;0.87) |
| Household income Q3 | 0.84(0.82;0.85) | 0.74(0.73;0.75) |
| Household income Q4 | 0.81(0.80;0.83) | 0.65(0.64;0.66) |
| Household income Q5 (highest) | 0.78(0.76;0.79) | 0.61(0.60;0.63) |
| Stable medium:Cohort 2 | 1.04(0.99;1.10) | 1.03(0.98;1.07) |
| Stable low:Cohort 2 | 1.02(0.96;1.08) | 0.98(0.94;1.03) |
| Improving to high:Cohort 2 | 1.08(0.96;1.20) | 1.04(0.94;1.14) |
| Improving to medium:Cohort 2 | 0.99(0.89;1.10) | 1.03(0.94;1.13) |
| Declining to medium:Cohort 2 | 1.04(0.96;1.13) | 1.00(0.92;1.07) |
| Declining to low:Cohort 2 | 0.97(0.89;1.06) | 0.96(0.89;1.04) |
| Stable medium:Cohort 3 | 1.03(0.98;1.09) | 0.98(0.93;1.02) |
| Stable low:Cohort 3 | 1.00(0.94;1.06) | 0.93(0.89;0.98) |
| Improving to high:Cohort 3 | 1.03(0.93;1.13) | 0.99(0.91;1.08) |
| Improving to medium:Cohort 3 | 0.95(0.87;1.04) | 1.00(0.92;1.08) |
| Declining to medium:Cohort 3 | 1.05(0.94;1.16) | 0.94(0.85;1.04) |
| Declining to low:Cohort 3 | 0.94(0.86;1.03) | 0.88(0.81;0.95) |
| Stable medium:Cohort 4 | 1.03(0.98;1.09) | 0.96(0.92;1.00) |
| Stable low:Cohort 4 | 1.01(0.96;1.07) | 0.91(0.87;0.96) |
| Improving to high:Cohort 4 | 0.99(0.88;1.12) | 1.01(0.91;1.12) |
| Improving to medium:Cohort 4 | 0.99(0.89;1.11) | 0.92(0.83;1.02) |
| Declining to medium:Cohort 4 | 1.03(0.93;1.13) | 0.98(0.89;1.07) |
| Declining to low:Cohort 4 | 0.98(0.89;1.07) | 0.92(0.85;0.99) |
| Odds ratios (95% CI). Models adjusted for maternal age, parity, migration background (ethnicity and generation) and household income. For categorical variables, omitted categories are reference categories. | | |

**Supplementary table 9. Sensitivity analysis 7: Excluded observations with implausible birthweight given gestational age values (N= 2 328 351).**

| **Variable** | **Preterm birth** | **SGA** |
| --- | --- | --- |
| Stable medium | 1.04(0.99;1.09) | 1.03(0.99;1.07) |
| Stable low | 1.12(1.07;1.17) | 1.19(1.15;1.23) |
| Improving to high | 0.99(0.91;1.07) | 0.98(0.92;1.04) |
| Improving to medium | 1.09(1.01;1.17) | 1.04(0.98;1.10) |
| Declining to medium | 1.02(0.95;1.09) | 1.03(0.98;1.08) |
| Declining to low | 1.12(1.05;1.20) | 1.12(1.06;1.17) |
| Cohort 2 | 0.98(0.94;1.02) | 0.91(0.88;0.94) |
| Cohort 3 | 0.97(0.93;1.01) | 0.92(0.89;0.96) |
| Cohort 4 | 0.92(0.88;0.96) | 0.93(0.90;0.96) |
| Maternal age (20-34) | 1.02(0.97;1.07) | 0.97(0.94;1.00) |
| Maternal age (>35) | 1.21(1.16;1.27) | 1.14(1.10;1.18) |
| Multiparous | 0.52(0.51;0.52) | 0.46(0.45;0.46) |
| Moroccan | 0.79(0.76;0.82) | 0.92(0.90;0.94) |
| Turkish | 0.89(0.86;0.92) | 1.08(1.05;1.10) |
| Suriname | 1.42(1.37;1.47) | 2.42(2.37;2.48) |
| Antillean | 1.26(1.20;1.32) | 1.43(1.38;1.48) |
| Other non-western | 0.91(0.88;0.93) | 1.33(1.30;1.35) |
| Other western | 0.89(0.87;0.91) | 1.06(1.04;1.08) |
| Migrant first generation | 0.94(0.91;0.96) | 0.92(0.90;0.94) |
| Migrant second generation | 1.07(1.05;1.10) | 1.08(1.07;1.10) |
| Household income Q2 | 0.87(0.86;0.89) | 0.85(0.83;0.86) |
| Household income Q3 | 0.84(0.82;0.85) | 0.73(0.72;0.74) |
| Household income Q4 | 0.81(0.80;0.83) | 0.65(0.64;0.65) |
| Household income Q5 (highest) | 0.78(0.76;0.79) | 0.60(0.60;0.61) |
| Stable medium:Cohort 2 | 1.04(0.99;1.10) | 1.05(1.01;1.09) |
| Stable low:Cohort 2 | 1.02(0.96;1.08) | 1.01(0.97;1.05) |
| Improving to high:Cohort 2 | 1.08(0.96;1.20) | 1.04(0.95;1.13) |
| Improving to medium:Cohort 2 | 0.99(0.89;1.10) | 1.04(0.96;1.13) |
| Declining to medium:Cohort 2 | 1.04(0.96;1.13) | 0.99(0.93;1.06) |
| Declining to low:Cohort 2 | 0.97(0.89;1.06) | 0.98(0.92;1.05) |
| Stable medium:Cohort 3 | 1.03(0.98;1.09) | 1.00(0.96;1.04) |
| Stable low:Cohort 3 | 1.00(0.94;1.06) | 0.96(0.92;1.00) |
| Improving to high:Cohort 3 | 1.03(0.93;1.13) | 1.02(0.94;1.09) |
| Improving to medium:Cohort 3 | 0.95(0.87;1.04) | 1.04(0.98;1.12) |
| Declining to medium:Cohort 3 | 1.05(0.94;1.16) | 0.96(0.88;1.04) |
| Declining to low:Cohort 3 | 0.94(0.86;1.03) | 0.89(0.83;0.96) |
| Stable medium:Cohort 4 | 1.03(0.98;1.09) | 0.98(0.94;1.02) |
| Stable low:Cohort 4 | 1.01(0.96;1.07) | 0.95(0.91;0.99) |
| Improving to high:Cohort 4 | 0.99(0.88;1.12) | 1.04(0.95;1.14) |
| Improving to medium:Cohort 4 | 0.99(0.89;1.11) | 0.95(0.87;1.03) |
| Declining to medium:Cohort 4 | 1.03(0.93;1.13) | 0.97(0.90;1.05) |
| Declining to low:Cohort 4 | 0.98(0.89;1.07) | 0.95(0.89;1.01) |
| Odds ratios (95% CI). Models adjusted for maternal age, parity, migration background (ethnicity and generation) and household income.  For categorical variables, omitted categories are reference categories. | | |

**Supplementary table 10. Sensitivity analysis 8: Joint regression models for binary outcomes (N= 2 334 036).**

| **Variable** | **Preterm birth** | **SGA** |
| --- | --- | --- |
| Stable medium | 1.05(0.99;1.12) | 1.04(0.99;1.09) |
| Stable low | 1.10(1.04;1.16) | 1.19(1.15;1.23) |
| Improving to high | 1.02(0.94;1.11) | 0.99(0.93;1.06) |
| Improving to medium | 1.11(1.02;1.20) | 1.04(0.98;1.11) |
| Declining to medium | 1.03(0.96;1.10) | 1.03(0.98;1.08) |
| Declining to low | 1.11(1.02;1.20) | 1.11(1.06;1.17) |
| Cohort 2 | 1.01(0.96;1.06) | 0.92(0.88;0.95) |
| Cohort 3 | 1.01(0.96;1.06) | 0.94(0.90;0.97) |
| Cohort 4 | 0.95(0.90;0.99) | 0.95(0.92;0.98) |
| Maternal age (20-34) | 0.98(0.93;1.03) | 0.96(0.93;0.99) |
| Maternal age (>35) | 1.11(1.05;1.17) | 1.09(1.06;1.13) |
| Multiparous | 0.56(0.55;0.57) | 0.48(0.47;0.48) |
| Moroccan | 0.82(0.79;0.85) | 0.94(0.92;0.97) |
| Turkish | 0.89(0.86;0.93) | 1.09(1.07;1.12) |
| Suriname | 1.22(1.18;1.27) | 2.34(2.29;2.40) |
| Antillean | 1.21(1.15;1.28) | 1.38(1.33;1.43) |
| Other non-western | 0.87(0.85;0.90) | 1.34(1.32;1.37) |
| Other western | 0.90(0.88;0.92) | 1.08(1.06;1.10) |
| Migrant first generation | 0.94(0.91;0.96) | 0.92(0.90;0.94) |
| Migrant second generation | 1.07(1.04;1.10) | 1.08(1.06;1.10) |
| Household income Q2 | 0.89(0.88;0.91) | 0.85(0.84;0.87) |
| Household income Q3 | 0.88(0.86;0.90) | 0.74(0.73;0.75) |
| Household income Q4 | 0.87(0.85;0.89) | 0.66(0.65;0.67) |
| Household income Q5 (highest) | 0.85(0.83;0.87) | 0.62(0.61;0.63) |
| Stable medium:Cohort 2 | 1.00(0.95;1.06) | 1.03(0.99;1.07) |
| Stable low:Cohort 2 | 1.01(0.95;1.07) | 1.00(0.96;1.05) |
| Improving to high:Cohort 2 | 1.06(0.94;1.19) | 1.03(0.94;1.12) |
| Improving to medium:Cohort 2 | 0.97(0.87;1.09) | 1.05(0.96;1.14) |
| Declining to medium:Cohort 2 | 1.02(0.93;1.12) | 0.99(0.92;1.06) |
| Declining to low:Cohort 2 | 0.99(0.90;1.09) | 0.99(0.92;1.06) |
| Stable medium:Cohort 3 | 1.00(0.95;1.06) | 0.98(0.94;1.02) |
| Stable low:Cohort 3 | 0.98(0.92;1.05) | 0.95(0.91;0.99) |
| Improving to high:Cohort 3 | 0.99(0.90;1.10) | 1.00(0.92;1.08) |
| Improving to medium:Cohort 3 | 0.92(0.83;1.01) | 1.04(0.97;1.11) |
| Declining to medium:Cohort 3 | 1.05(0.94;1.18) | 0.95(0.87;1.03) |
| Declining to low:Cohort 3 | 0.94(0.85;1.04) | 0.88(0.82;0.95) |
| Stable medium:Cohort 4 | 1.01(0.95;1.06) | 0.96(0.93;1.00) |
| Stable low:Cohort 4 | 1.01(0.95;1.07) | 0.94(0.90;0.98) |
| Improving to high:Cohort 4 | 0.95(0.83;1.08) | 1.02(0.93;1.12) |
| Improving to medium:Cohort 4 | 0.98(0.87;1.10) | 0.94(0.87;1.03) |
| Declining to medium:Cohort 4 | 1.00(0.90;1.12) | 0.96(0.89;1.04) |
| Declining to low:Cohort 4 | 0.98(0.89;1.08) | 0.94(0.88;1.00) |
| Odds ratios (95% CI).  Models adjusted for maternal age, parity, migration background (ethnicity and generation) and household income. For categorical variables, omitted categories are reference categories.  Dependence parameter θ (95% CI)= 0.19(0.17-0.21). | | |

**Supplementary table 11. Sensitivity analysis 9: Siblings-comparison analysis (N= 2 334 036).**

| **Variable** | **Preterm birth** | **SGA** |
| --- | --- | --- |
| Stable medium | 1.07(0.99;1.15) | 1.07(0.99;1.20) |
| Stable low | 1.12(1.05;1.20) | 1.10(1.04;1.16) |
| Improving to high | 1.07(0.99;1.15) | 1.05(0.99;1.12) |
| Improving to medium | 1.11(1.03;1.21) | 1.07(0.98;1.16) |
| Declining to medium | 1.09(0.99;1.19) | 1.04(0.98;1.10) |
| Declining to low | 1.10(1.02;1.19) | 1.06(1.01;1.12) |
| Cohort 2 | 0.71(0.69;0.73) | 0.59(0.57;0.60) |
| Cohort 3 | 0.71(0.51;0.55) | 0.36(0.35;0.37) |
| Cohort 4 | 0.36(0.34;0.38) | 0.22(0.21;0.23) |
| Maternal age (20-34) | 1.06(0.97;1.17) | 1.01(0.94;1.09) |
| Maternal age (>35) | 0.98(0.88;1.09) | 0.83(0.77;0.90) |
| Multiparous | 0.51(0.51;0.52) | 0.47(0.47;0.48) |
| Household income Q2 | 0.95(0.87;0.95) | 0.95(0.92;0.98) |
| Household income Q3 | 1.05(1.00;1.10) | 1.09(1.05;1.13) |
| Household income Q4 | 1.31(1.25;1.38) | 1.35(1.29;1.40) |
| Household income Q5 (highest) | 1.81(1.70;1.92) | 1.73(1.65;1.82) |
| Stable medium:Cohort 2 | 1.04(0.99;1.10) | 1.03(0.98;1.07) |
| Stable low:Cohort 2 | 1.02(0.96;1.08) | 0.98(0.94;1.03) |
| Improving to high:Cohort 2 | 1.08(0.96;1.20) | 1.04(0.94;1.14) |
| Improving to medium:Cohort 2 | 0.99(0.89;1.1) | 1.03(0.94;1.13) |
| Declining to medium:Cohort 2 | 1.04(0.96;1.13) | 1.00(0.92;1.07) |
| Declining to low:Cohort 2 | 0.97(0.89;1.06) | 0.96(0.89;1.04) |
| Stable medium:Cohort 3 | 1.03(0.98;1.09) | 0.98(0.93;1.02) |
| Stable low:Cohort 3 | 1.00(0.94;1.06) | 0.93(0.89;0.98) |
| Improving to high:Cohort 3 | 1.03(0.93;1.13) | 0.99(0.91;1.08) |
| Improving to medium:Cohort 3 | 0.95(0.87;1.04) | 1.00(0.92;1.08) |
| Declining to medium:Cohort 3 | 1.05(0.94;1.16) | 0.94(0.85;1.04) |
| Declining to low:Cohort 3 | 0.94(0.86;1.03) | 0.88(0.81;0.95) |
| Stable medium:Cohort 4 | 1.03(0.98;1.09) | 0.96(0.92;1.00) |
| Stable low:Cohort 4 | 1.01(0.96;1.07) | 0.91(0.87;0.96) |
| Improving to high:Cohort 4 | 0.99(0.88;1.12) | 1.01(0.91;1.12) |
| Improving to medium:Cohort 4 | 0.99(0.89;1.11) | 0.92(0.83;1.02) |
| Declining to medium:Cohort 4 | 1.03(0.93;1.13) | 0.98(0.89;1.07) |
| Declining to low:Cohort 4 | 0.98(0.89;1.07) | 0.92(0.85;0.99) |
| Odds ratios (95% CI). Models adjusted for time-varying covariates: maternal age, parity, and household income. For categorical variables, omitted categories are reference categories. Dependence parameter θ (95% CI)= 0.19(0.17-0.21). | | |

**Supplementary figure 1. Alluvial diagram showing changes in neighbourhood socioeconomic status (SES) during 4-years periods in the Netherlands (1998-2014)**


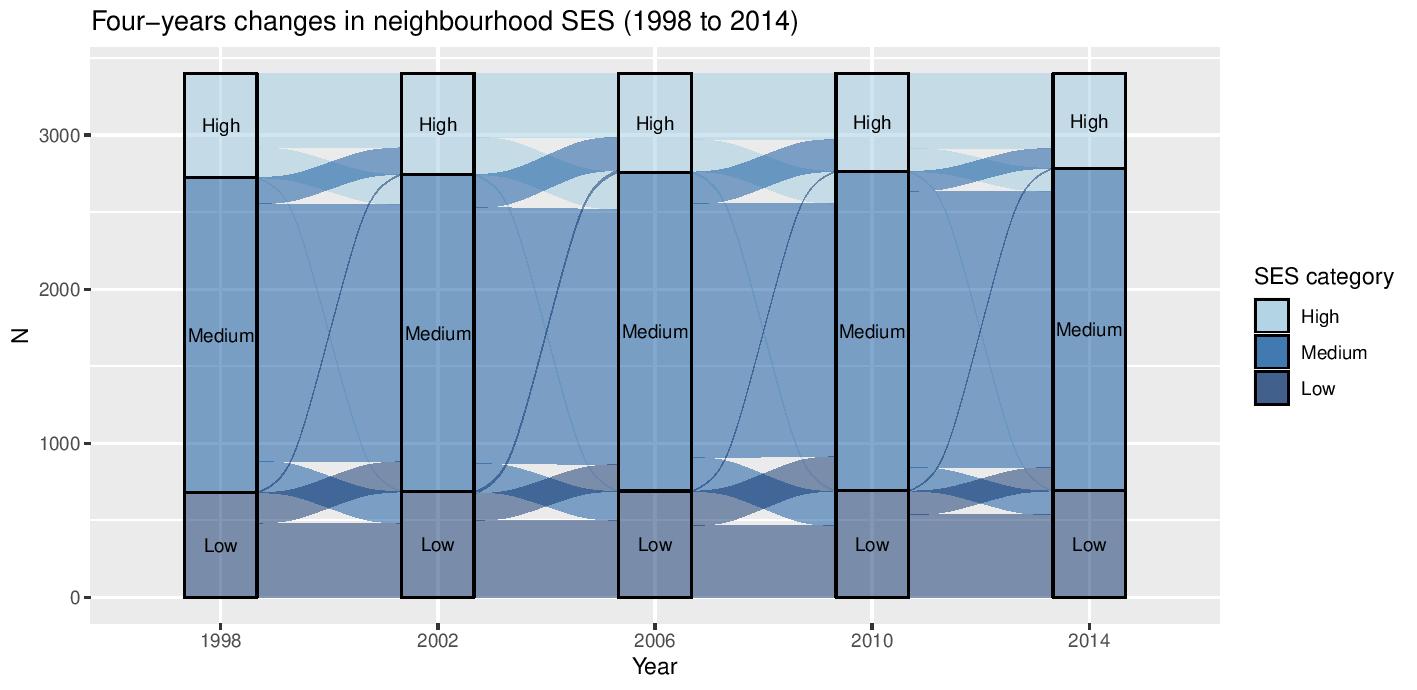


**Supplementary Figure 2. Changes in the relationship between neighbourhood SES trajectories and birth outcomes across birth periods 1 (2003-2005) and 4 (2014-2017).**


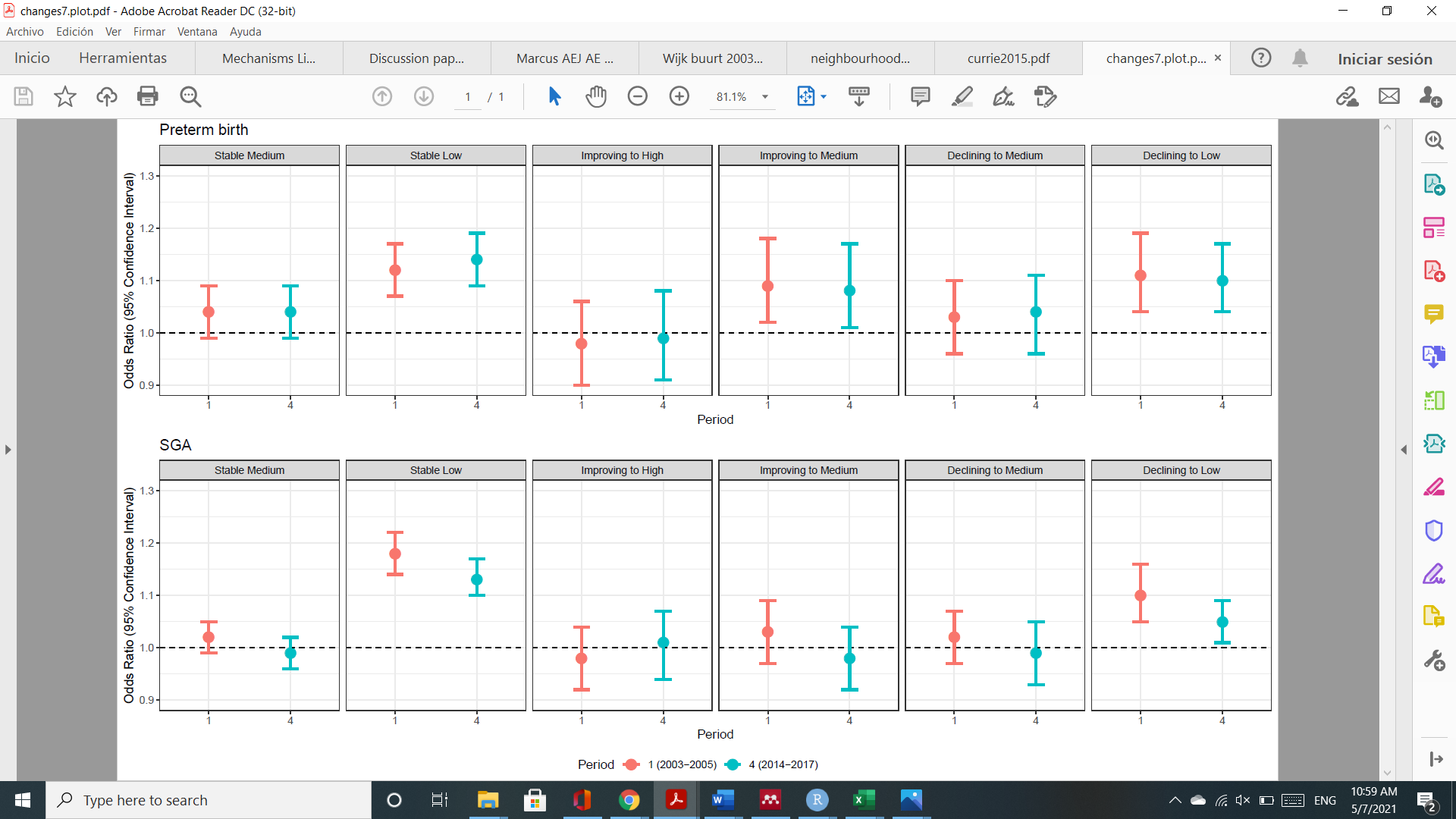

Supplement: Supplementary file 1 [file mmc1.docx]
